# SUPPRESSION OF MICROBIOME SENSING—FREE FATTY ACID RECEPTOR 2 (FFAR2) SIGNALING EXHIBITS EARLY AGING

**DOI:** 10.1093/geroni/igad104.1815

**Published:** 2023-12-21

**Authors:** Sidharth Mishra, Santosh Kumar Prajapati, Shalini Jain, Hariom Yadav

**Affiliations:** University of South Florida, Tampa, Florida, United States; University of South Florida, Tampa, Florida, United States; University of South Florida, Tampa, Florida, United States; University of South Florida, Tampa, Florida, United States

## Abstract

Abnormalities in gut microbiome contribute to age-related diseases, causal to unhealthy aging, and early mortality, but mechanisms still remain largely unknown. Our recent research shows that old microbiota lacks the capacity to produce beneficial metabolites like short-chain fatty acids (SCFAs), which in turn promote gut permeability and systemic inflammation. SCFAs activates free fatty acid receptor 2 and 3 (FFAR2/3) signaling, at least in part, to exhibit their benefits on host cells; however, the role of these signaling in aging process is not well understood. We discovered that in addition to gut the brains of old mice show lower expression of FFAR2. Further, FFAR2 deletion in the intestine shows a shorter life span compared to wild-type mice. They also show a higher cognitive decline at early age. Similarly, intestine specific FFAR2 KO in APP/PS1 (Alzheimer’s disease [AD] model) background shows higher progression of AD including amyloid beta (A-beta) and tau accumulation, linked with higher blood brain barrier (BBB) permeability and neuroinflammation. These changes are associated with increased gut permeability and systemic inflammaging. Interestingly, a newly discovered natural FFAR2 agonist- Fenchol reduces these abnormalities in gut-brain and prevented the progression of cognitive decline and AD pathology. These results show that microbiome sensing FFAR2 signaling deficiency develops an early aging phenotype in mice, while its agonism using natural compound can delay/ prevent the progression of early aging.

